# Omega-3 fatty acid supplementation affects tryptophan metabolism during a 12-week endurance training in amateur runners: a randomized controlled trial

**DOI:** 10.1038/s41598-024-54112-x

**Published:** 2024-02-19

**Authors:** Maja Tomczyk, Monika Bidzan-Wiącek, Jakub Antoni Kortas, Magdalena Kochanowicz, Zbigniew Jost, Helena L. Fisk, Philip C. Calder, Jędrzej Antosiewicz

**Affiliations:** 1 Department of Biochemistry, Gdansk University of Physical Education and Sport, 80-336 Gdansk, Poland; 2 Department of Bioenergetics and Physiology of Exercise, Medical University of Gdansk, 80-211 Gdansk, Poland; 3 Department of Biomechanics and Sports Engineering, Gdansk University of Physical Education and Sport, 80-336 Gdansk, Poland; 4 Department of Physiotherapy, Medical University of Gdansk, 80-211 Gdansk, Poland; 5 School of Human Development and Health, Faculty of Medicine, University of Southampton, Southampton, SO16 6YD UK; 6 NIHR Southampton Biomedical Research Centre, University Hospital Southampton NHS Foundation Trust and University of Southampton, Southampton, SO16 6YD UK

**Keywords:** Omega-3 fatty acids, Endurance training, Tryptophan metabolism, 3-hydroxykynurenine, Picolinic acid, Biochemistry, Biomarkers

## Abstract

The effects of long-term omega-3 polyunsaturated fatty acid (n-3 PUFA) supplementation during endurance training on tryptophan (Trp) metabolism and mental state of healthy individuals have not been evaluated so far. Concentrations of plasma Trp, its metabolites and IL-6 were assessed in 26 male runners before and after a 12-week training program combined with supplementation of n-3 PUFAs (O-3 + TRAIN group) or medium chain triglycerides (MCTs; TRAIN group). After the 12-week program participants' mood before and after stress induction was also assessed. The effects of the same supplementation protocol were evaluated also in 14 inactive subjects (O-3 + SEDEN group). Concentrations of 3-hydroxykynurenine (3-HK) and picolinic acid (PA) significantly increased only in the O-3 + TRAIN group (*p* = 0.01; ${\eta }_{p}^{2}$ = 0.22 and *p* = 0.01; ${\eta }_{p }^{2}$= 0.26). Favorable, but not statistically significant changes in the concentrations of kynurenic acid (KYNA) (*p* = 0.06; ${\eta }_{p }^{2}$= 0.14), xanthurenic acid (XA) (*p* = 0.07; ${\eta }_{p }^{2}$= 0.13) and 3-hydroxyanthranilic acid (3-HAA) (*p* = 0.06; ${\eta }_{p }^{2}$= 0.15) and in the ratio of neurotoxic to neuroprotective metabolites were seen also only in the O-3 + TRAIN group. No changes in mood and IL-6 concentrations were observed in either group. Supplementation with n-3 PUFAs during endurance training has beneficial effects on Trp's neuroprotective metabolites.

*Trial registry*: This study was registered at ClinicalTrials.gov with identifier NCT05520437 (14/07/2021 first trial registration and 2018/31/N/NZ7/02962 second trial registration).

## Introduction

There is an increasing number of studies demonstrating that insufficiency of omega-3 polyunsaturated fatty acids (n-3 PUFAs), especially eicosapentaenoic acid (EPA) and docosahexaenoic acid (DHA) is common in many countries and regions of the world^1–3^. Improvement in n-3 PUFA status exerts multidirectional health benefits, including reduced inflammation, which is also a well-described effect of engaging in physical activity^4–7^. It is also known that especially endurance exercise modulates tryptophan (Trp) metabolism^8–10^. Trp is an essential amino acid required for several metabolic pathways; its conversion into kynurenine (KYN) is the major fate, consuming around 95% of Trp^11^. KYN can be converted into kynurenic acid (KYNA), 3-hydroxyanthranilic acid (3-HAA), quinolinic acid (QA), 3-hydroxykynurenine (3-HK), xanthurenic acid (XA), picolinic acid (PA) and others^12^. Some of these metabolites, such as KYN, 3-HK, XA and PA can cross the blood–brain barrier, while QA, KYNA and 3-HAA cannot^13,14^. In vitro and in vivo studies have shown that among these, KYNA, XA and PA have neuroprotective and dopamine-stimulating effects^13,15^. Studies in rodents show that adaptation to endurance exercise is associated with increased expression of genes encoding KATs, enzymes responsible for the transformation of KYN into KYNA^16^. This is in line with human data which show an increase in plasma levels KYNA and QA after marathon running in healthy adults^17^. However, data on changes in the concentrations of a range of Trp metabolites following long-term endurance training in healthy individuals are scarce. N-3 PUFAs appear to be another promising factor modulating Trp metabolism: yet, there is only one study showing that deficiency in n-3 PUFAs in rodents was associated with increased KYN levels in the hippocampus^18^. Nonetheless, neither effect of long-term n-3 PUFA supplementation nor it’s combination with exercise training on Trp metabolism in humans has not been studied so far. In addition, there are rationales that both n-3 PUFA supplementation and physical activity, through modulation of the Trp pathway, may influence a range of psychopathologic disorders^19,20^. However, the effects of this combination in the long-term in healthy individuals are unknown.

The aim of the present study was to evaluate whether 12-weeks of n-3 PUFA supplementation would demonstrate an additive effect on alterations in Trp and its metabolites when combined with endurance training in male amateur endurance runners. For comparison, the effects of the same supplementation protocol were evaluated in 14 inactive subjects. Changes in ratio of neurotoxic to neuroprotective Trp metabolites and IL-6 concentration were also investigated as well as participants' mood before and after stress induction following the 12-week training program.

## Methods

### Study design

The study was approved by the Bioethics Committee for Research Projects at the University of Gdańsk (protocol number 44/2020) and was conducted in accordance with the Declaration of Helsinki. All the participants provided written informed consent prior to the study procedures and anonymity and confidentiality were ensured by replacing the participants personal identification with a code. This study is part of a training and supplementation program in long-distance runners conducted in the Laboratory of Physical Exercise and Department of Biochemistry of the Academy of Physical Education and Sport in Gdansk, which is described in detail elsewhere^21^. Briefly, the randomized experimental study lasted 12 weeks, during which time amateur male endurance runners performed progressive endurance training supervised by a track and field coach. Throughout the entire cycle, participants took supplementation with n-3 PUFAs (O-3 group; n = 14; 2234 mg of EPA and 930 mg of DHA daily) or medium chain triglycerides (MCTs) (CON group; n = 12; 4000 mg of MCT daily) on the basis of a previous random allocation using an online randomizer (http://www.randomizer.org). Before the beginning and after the completion of the program, an exercise performance test took place. Moreover, blood samples were collected under resting conditions for determination of n-3 PUFAs and Trp metabolites.

### Participants

40 amateur male endurance runners were recruited through advertisements on the internet. None of the participants had chronic diseases, smoked, took medications or dietary supplements, including n-3 PUFAs. All participants agreed to carry out only the training courses included in the program and to keep their diet as constant as possible during the experimental period. Participants excluded from the final analysis completed insufficient training sessions (< 80%) or withdrew from the study for health or personal reasons. From 40 participants enrolled, 26 completed the entire study. Characteristics of those participants and their total daily energy and macronutrient intake during the intervention are shown in Table 1.

**Table 1:** Baseline characteristics of trained participants and macronutrient intake during the intervention.

Variable	O-3 + TRAIN (n = 14)	TRAIN (n = 12)
	Mean ± SD	Mean ± SD
Age (y)	37 ± 3	37 ± 4
Body mass (kg)	76 ± 11	78 ± 8
Height (cm)	181 ± 7	180 ± 4
VO_2peak_ (mL*kg^−1^*min^−1^)	53.6 ± 4	54.7 ± 7
HRmax (beats min^−1^)	190 ± 9	186 ± 9
Total daily energy and macronutrient intake during the intervention		
Energy (kcal)	Pre 2393 ± 453	Pre 2456 ± 587
	End 2429 ± 420	End 2338 ± 627
Carbohydrate (g)	Pre 300.85 ± 62.63	Pre 310.17 ± 110.79
	End 289.08 ± 45.90	End 301.58 ± 126.95
Protein (g)	Pre 97.54 ± 20.14	Pre 99.08 ± 19.85
	End 102.46 ± 17.03	End 94.75 ± 17.11
Fat (g)	Pre 83.38 ± 26.95	Pre 86.42 ± 18.0
	End 91.69 ± 27.16*	End 79.0 ± 15.5

Data are presented as mean ± SD *statistically significant difference in groups (Δ) with a trend of higher intake in the O3 + TRAIN group and lower intake in the TRAIN group.

The control group included 11 physically inactive males (O-3 group + SEDEN; 38 ± 6 y, 180 ± 6 cm, 87 ± 9 kg). The majority of them had sedentary office-based jobs and the maximum amount of moderate physical activity per week was 120 min. Exclusion criteria included DSM-5psychiatric disorders other than depression and anxiety (American Psychiatric Association, 2013), neurological disorders, severe chronic conditions, and use of dietary supplements containing n-3 PUFAs.

### Training characteristics

Participants underwent twelve weeks of structured endurance training under the supervision of a track and field coach as previously described^21^. The participants performed running training and functional training three times and once a week, respectively. Training intensity was established according to the heart rate values corresponding to the appropriate ventilation thresholds. Participants trained in moderate, heavy, and severe intensity zones (Z1, Z2, Z3); moreover, the time spent in these zones was respectively ~ 80%, ~ 15% and ~ 5%. The weekly training volume was approximately 31 km for both groups. However, in the last week, a volume reduction was used for psychophysical recovery. All activities were monitored by a Polar M430 wristwatch and a H9 heart rate sensor.

### Sample collection

Blood samples were collected into 4 mL sodium citrate vacutainer tubes and centrifuged at 4 °C (4000 × g for 10 min). After that, plasma and RBCs were collected with a disposable Pasteur pipette and transferred into separate Eppendorf probes and stored in a − 80 °C freezer until further analysis.

### Assessment of RBC EPA and DHA

Determinations of RBC EPA and DHA were performed according to the method by Fisk et al.^22^. Plasma and RBC lipids were extracted into chloroform: methanol and fatty acid methyl esters were formed by heating the lipid extract with methanolic sulphuric acid. The fatty acid methyl esters were separated by gas chromatography on a Hewlett Packard 6890 gas chromatograph fitted with a BPX-70 column. Fatty acid methyl esters were identified by comparison with runtimes of authentic standards and data were expressed as weight % of total fatty acids.

### Assessment of tryptophan, its metabolites and IL-6

Determinations of plasma concentrations of Trp and its metabolites (3-HK, KYN, KYNA, QA, XA, PA, 3-HAA) were performed using high-performance liquid chromatography with tandem mass spectrometry (LC–MS/MS), with prior protein precipitation and derivatization. To 50 µL of plasma pipetted into deep well plate (polypropylene, U-bottom, 1 mL well volume), 250 µL of acetonitrilic solution of internal standards was added. After covering the plate with adhesive foil, it was mixed by vortexing (1100 rpm, RT, 30 min) followed by centrifugation (3000 rpm, 916 rcf, RT, 10 min). 50 µL of supernatant was transferred to a new plate (polypropylene, U-bottom, 300 µL well volume) and dried in an air flow (10 min, 55 °C). Then 50 µL of 3 M methanolic solution of hydrochloric acid was added. After covering the plate with adhesive foil, incubation took place (25 min, 60 °C); thereafter the sample was dried in an air flow (10 min, 55 °C). To the dry residue, 100 µL of 0.1% aqueous formic acid solution was added. The whole solution was mixed by vortexing (5 min, 350 rpm) and injected into an ExionLC™ (Sciex, Framingham, MA, USA) LC chromatographic system equipped with two binary pumps, degasser, column oven and PAL HTC autosampler (CTC Analytics AG, Zwinger, Switzerland), coupled with 4500 QTrap (Sciex) triple quadrupole mass spectrometer. Plasma concentrations of IL-6 was measured using sandwich ELISA kits (R&D Systems, Minneapolis, MN, USA) according to the manufacturer’s protocol (catalog no. HS600B).

### Mood assessment procedure

After completion of the 12-week program, mood was assessed in all participants using a validated psychological measure, the Mood Adjective Checklist (UMACL)^23,24^. Subsequently, following an incremental test to exhaustion on a motorized treadmill, a stress response was elicited using a validated stress manipulation test, the Trier Social Stress Test (TSST)^25^. After the stress manipulation test, mood was reassessed. In the control group, mood was assessed after completion of 12-weeks supplementation period, which was followed by stress induction and mood reassessment. The Polish adaptation of the Mood Adjective Check List (UMACL)^23,24^ was used to assess mood. The questionnaire comprises a list of 29 adjectives. Participants rate the degree to which their present mood corresponds to each of the adjectives on a 1 to 4 scale. The final score is represented by the three dimensions: energetic arousal (EA), tense arousal (TA) and hedonic tone (HT). High levels of energetic arousal correspond to being restful, energetic and vigorous; high scores of tense arousal correspond to being stressed, anxious or tense; and high scores of hedonic tone are associated with being cheerful, satisfied and happy. Trier Social Stress Test (TSST) was used to induce a stress response in participants^25^. The TSST is a three-stage psychosocial stress task conducted in front of a panel of experimenters, (i) preparation; (ii) public speaking task; and (iii) mental arithmetic task. The TSST is a reliable method for inducing psychosocial stress^26^.

### Statistical analysis

Data are given as means with standard deviations (SD). Statistical analyses were performed using the Statistica 13.1 software package. Shapiro–Wilk test was used to assess the homogeneity of dispersion from normal distribution. In the first part of the analyses, paired tests were performed to identify differences between the groups. Brown–Forsythe test was used to evaluate the homogeneity of variance. Then, separate 2 (group: O-3, CON) × 2 (time: PRE, POST) repeated measures analyses of variance (rANOVA) was used. For a homogenous sample, the analysis of variance (ANOVA) for repeated measures and a post hoc Tukey’s test for unequal sample sizes were performed to identify significantly different results. For a heterogeneous sample, ANOVA Friedman test and Dunn–Bonferroni post hoc test were used. To estimate interaction effect sizes, partial eta squared $({\eta }_{p}^{2}$) was computed with ${\eta }_{p}^{2}\ge 0.01$ indicating small, $\ge$ 0.059 medium and $\ge$ 0.138 large effects. The relationships between variables were evaluated using the Spearman correlation coefficient. The sample size was predetermined by using a power calculation in G ∗ power v3.1.9.7 software. Based on a priori power analysis for family F tests in ANOVA repeated measures, within-between interaction, at least 12 participants were included in each group (α = 0.05, 1–β = 0.8, f = 0.25, r_rm_ = 0.85; ε = 1). Finally, 20 participants were recruited in each group to account for an losses. The level of significance was set at *p* < 0.05.

## Results

No notable differences in characteristics of trained participants at study entry or in energy, carbohydrate and protein intake during the intervention were observed within either group (Table 1) (O-3 + TRAIN group: *p* > 0.99, *p* = 0.54, *p* = 0.58; TRAIN group: *p* = 0.20, *p* > 0.99, *p* = 0.77 for energy and each macronutrient, respectively). However, significantly higher fat intake was found in the O-3 + TRAIN group compared to the TRAIN group at the end of the study (*p* = 0.032).

**Table 2:** EPA, DHA and their sum as a percentage of total fatty acids in red blood cells and plasma.

	O-3 + TRAIN (n = 14)			TRAIN (n = 12)			rANOVA
	PRE	POST	Δ (CI)	PRE	POST	Δ (CI)	Group x time
DHA (% in red blood cells)	4.68 ± 1.03	6.69 ± 0.76*	2.01 (1.66;2.35)	4.42 ± 1.11	4.68 ± 1.01	0.2 (− 0.28;0.68)	< 0.01
EPA (% in red blood cells)	1.11 ± 0.39	4.88 ± 1.11*	3.77 (3.14;4.41)	1.16 ± 0.3	1.18 ± 0.44	− 0.01 (− 0.28;0.26)	< 0.01
DHA (% in plasma)	2.38 ± 0.5	4.01 ± 0.5*	1.64 (1.32;1.96)	2.18 ± 0.69	2.2 ± 0.78	− 0.09 (− 0.59;0.4)	< 0.01
EPA (% in plasma)	1.17 ± 0.51	5.15 ± 1.53*	3.94 (3.05;4.83)	1.19 ± 0.38	1.04 ± 0.46	-0.21 (− 0.61;0.2)	< 0.01
DHA + EPA (% in red blood cells)	5.79 ± 1.35	11.57 ± 1.7*	5.78 (4.9;6.66)	5.67 ± 1.37	5.86 ± 1.39	0.19 (− 0.5;0.88)	< 0.01
DHA + EPA (% in plasma)	3.59 ± 0.97	9.16 ± 1.97*	5.58 (4.43;6.73)	3.38 ± 0.97	3.08 ± 1.14	− 0.3 (− 1.16;0.56)	< 0.01

Values are presented as mean ± SD; EPA—eicosapentaenoic acid; DHA—docosahexaenoic acid. Δ—POST to PRE changes; CI—confidence interval of changes; rANOVA—repeated measurement analysis of variance*-statistically significant difference compared to PRE; p < 0.05.

Data are presented as mean ± SD *statistically significant difference in groups (Δ) with a trend of higher percentages in the O3 + TRAIN group and lower percentages in the TRAIN group.

DHA, EPA and their sum as a percentage of total fatty acids in red blood cells and plasma are provided in Table 2. A statistically significant increase in each parameter were observed in O-3 + TRAIN group (all *p* < 0.01) with no such changes in TRAIN group.

**Table 3:** The effect of n-3 PUFA supplementation combined with 12-week structured running training on plasma Trp metabolite concentrations in trained participants.

	O-3 + TRAIN (n = 14)			TRAIN (n = 12)			rANOVA p (${{\varvec{\eta}}}_{{\varvec{p}}}^{2})$
	PRE	POST	Δ (CI)	PRE	POST	Δ (CI)	Group x time (eta squared)
3-HK (ng/mL)	4.73 ± 1.33	5.09 ± 1.73*	0.36 (0.06;0.67)	4.54 ± 0.71	4.32 ± 0.5	− 0.22 (− 0.56;0.15)	**0.01 (0.22)**
KYN (ng/mL)	398.69 ± 53.57	403.42 ± 42.4	4.73 (− 27.75;37.21)	395.74 ± 56.65	382.48 ± 40.86	− 17.26 (− 52.1;17.59)	0.32 (0.04)
KYNA (ng/mL)	7.56 ± 1.82	8.27 ± 1.78	0.71 (− 0.01;1.43)	8.75 ± 2.53	7.8 ± 1.95	− 0.73 (− 2.27;0.81)	0.06 (0.14)
QA (ng/mL)	53.65 ± 14.35	47.44 ± 9.22	− 6.2 (− 14.64;2.23)	44.13 ± 8.89	42.98 ± 10.72	− 2.09 (− 7.55;3.36)	0.40 (0.03)
XA (ng/mL)	3.72 ± 1.85	4.38 ± 3.02	0.66 (− 0.23;1.55)	4.08 ± 1.22	3.48 ± 1.48	− 0.52 (− 1.59;0.55)	0.07 (0.13)
PA (ng/mL)	4.5 ± 1.72	5.8 ± 1.81*	1.3 (0.83;1.76)	5.12 ± 1.47	5.31 ± 1.58	0.23 (− 0.45;0.91)	**0.01 (0.26)**
3-HAA (ng/mL)	4.62 ± 1.7	5.04 ± 1.7	0.42 (− 0.08;0.92)	4.69 ± 2.01	4.1 ± 1.85	− 0.61 (-1.53;0.3)	0.06 (0.15)
Trp (nmol/mL)	55.35 ± 7.98	55.34 ± 8.38	− 0.01 (− 4.17; 4.15)	57.76 ± 8.44	57.95 ± 10.31	0.19 (− 9,84;10.22)	0.96 (0.00)

Values are presented as mean ± SD; 3-HK—3-hydroxykynurenine; KYN—kynurenine; KYNA—kynurenine acid; QA—Quinolinic acid; ; XA—xanthurenic acid; PA—picolinic acid; 3-HAA—3-Hydroxyanthranilic acid; Trp—tryptophan; Δ—POST to PRE changes; CI—confidence interval of changes; rANOVA—repeated measurement analysis of variance; *—statistically significant difference compared to PRE; *p* < 0.05.

**Figure 1:**
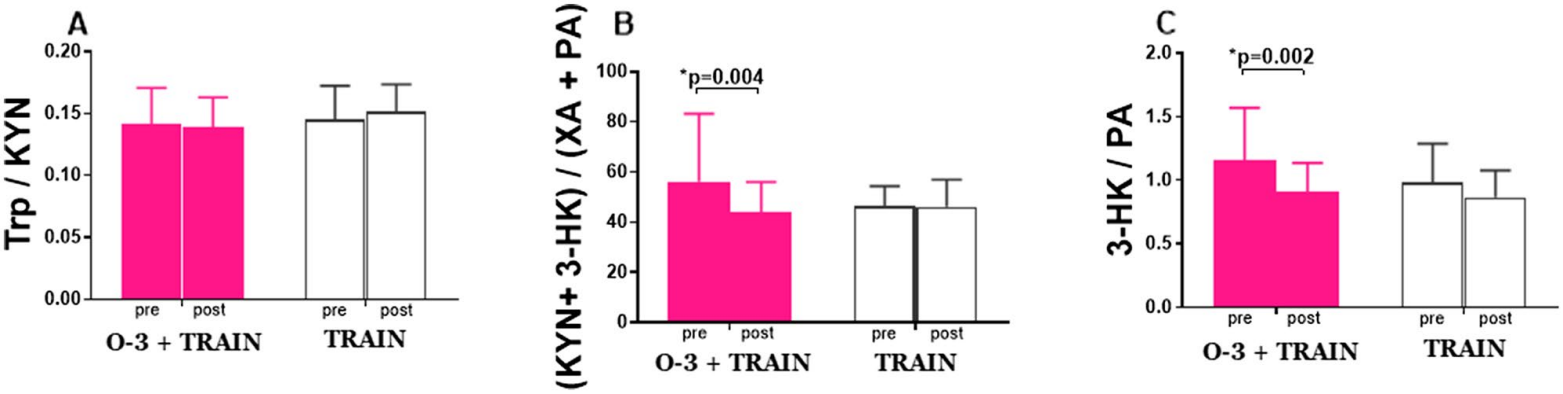
Plasma ratios Trp/KYN (**A**), (KYN + 3-HK)/(XA + PA) (**B**), and 3-HK/PA (**C**) under resting conditions (pre) and after 12-week training program (post) for supplementations groups: O-3 + TRAIN group (2234 mg of EPA and 930 mg of DHA daily) and TRAIN group (4000 mg of MCT daily). Trp—tryptophan; KYN—kynurenine; 3-HK—3-hydroxykynurenine; XA—xanthaurenic acid; PA—picolinic acid.

The plasma levels of Trp metabolites for both groups are provided in Table 3. A significant and large group x time interaction was observed for 3-HK (*p* = 0.01; ${\eta }_{p}^{2}$ = 0.22) where post hoc comparisons indicated a significant increase in the O-3 + TRAIN group (0.36 ng/mL, *p* = 0.02), with no change in TRAIN group (− 0.22 ng/mL, *p* = 0.21). A statistically significant increase was also noticed for PA in the O-3 + TRAIN group (*p* = 0.01; ${\eta }_{p }^{2}$= 0.26; post hoc O-3 + TRAIN: 1.3 ng/mL, *p* < 0.01 and CON: 0.23 ng/mL, *p* = 0.83). Large group x time interactions were observed for KYNA (${\eta }_{p }^{2}$= 0.14) and 3-HAA (${\eta }_{p}^{2}$= 0.15), medium group x time interaction was seen for XA (${\eta }_{p}^{2}$= 0.13) but without any statistical significance (*p* = 0.06, *p* = 0.06 and *p* = 0.07, respectively). The plasma ratio of (KYN + 3-HK)/(XA + PA) (Fig. 1B) significantly decreased in the O-3 + TRAIN group (Z = 2.86, *p* = 0.004).

Moreover, the ratio of 3-HK/PA was also significantly decreased after n-3 PUFA supplementation (Fig. 1C) (Z = 3.11, *p* = 0.002). However, no statistically significant intergroup difference was found (F = 3.8, *p* = 0.06 and F = 1.5, *p* = 0.23, respectively).

**Figure 2:**
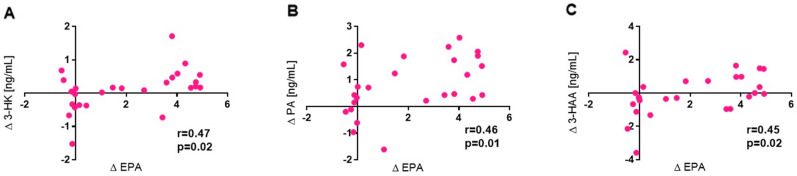
Correlations between changes in EPA (% of total fatty acids in red blood cells) and in 3-HK, PA and. 3-HAA under resting conditions.

Using data from all participants, the change in RBC EPA was positively correlated with changes in plasma 3-HK (r = 0.47, *p* = 0.02), PA (r = 0.46, *p* = 0.01) and 3-HAA (r = 0.45, *p* = 0.02) (Fig. 2). Additionally, in the control group there were significant positive correlations between changes in xanthurenic acid and baseline level of EPA (% of total fatty acids in RBCs), baseline DHA (% of total fatty acids in plasma) and baseline EPA (% of total fatty acids in plasma) (respectively: r = 0.56, r = 0.65, r = 0.71, all *p* < 0.05). There were no significant intergroup changes in participants' mood before and after stress induction (Table 4). Neither were there changes in the plasma concentration of the pro-inflammatory cytokine interleukin 6 (IL-6) in either group (O-3 + TRAIN: − 0.39 (− 0.9; 0.11); TRAIN: − 0.46–2.27; 1.35; *p* = 0.94).

**Table 4:** Scores on the three dimensions of the Mood Adjective Check List before and after stress induction.

	O-3 + TRAIN (n = 14)			TRAIN (n = 12)			rANOVA
	Before stress induction	After stress induction	Δ (CI)	Before stress induction	After stress induction	Δ (CI)	Group x time (eta squared)
Tense Arousal	14.79 ± 3.87	14.6 ± 4.06	− 0.5 (− 1.47;0.47)	15.09 ± 2.88	14.88 ± 3.44	− 0.5 (− 2.82;1.82)	0.99 (0.00)
Energetic Arousal	32.29 ± 4.32	30.2 ± 3.85	− 1.6 (− 4.64;1.44)	32.55 ± 4.23	32.88 ± 5.19	− 0.75 (− 3.46;1.96)	0.65 (0.01)
Hedonic Tone	26.79 ± 2.12	34.1 ± 2.92	7.2 (5.78;8.62)	27.36 ± 2.46	34 ± 4.87	6.88 (2.35;11.4)	0.86 (0.00)

Values are presented as mean ± SD; Δ—After stress induction to Before stress induction changes; CI—confidence interval of changes; rANOVA—repeated measurement analysis of variance.

### Control group

The second part of the analysis included the impact of n-3 PUFAs at the same dosage and length of supplementation on physically inactive individuals. A significant increase in plasma EPA and DHA concentrations was observed after 12 weeks of supplementation (*p* < 0.01, Table 5). However, there were no significant changes in the concentrations of Trp or it’s metabolites. Additionally, participants' mood before and after stress induction was assessed after 12 weeks of supplementation. The scores on the three dimensions of the Mood Adjective Check List scale were not significantly different before and after stress induction, i.e. Tense Arousal (18.5 ± 6.57 vs 16.07 ± 4.78, p = 0.74), Energetic Arousal (29.14 ± 6.71 vs 31.71 ± 4.66, p = 0.11), and Hedonic Tone (31.79 ± 4.46 vs 31.86 ± 4.64, p = 0.78).

**Table 5:** The effect of n-3 PUFA supplementation on plasma EPA, DHA and Trp metabolite concentrations in inactive participants.

	O-3 + SEDEN group (n = 11)		
	PRE	POST	*p*
EPA (% in plasma)	1.00 ± 0.68	4.77 ± 1.3*	** < 0.01**
DHA (% in plasma)	1.42 ± 0.74	3.15 ± 0.83*	** < 0.01**
EPA + DHA (% in plasma)	2.42 ± 1.39	7.92 ± 2.05*	** < 0.01**
3-HK (ng/mL)	5.14 ± 0.84	5.18 ± 1.13	0.77
KYN (ng/mL)	460.58 ± 70.57	459.39 ± 76.03	0.70
KYNA(ng/mL)	9.39 ± 3.35	10.05 ± 5.14	0.83
QA (ng/mL)	59.95 ± 15.08	62.53 ± 17.32	0.98
XA (ng/mL)	4.73 ± 1.99	5.01 ± 3.43	0.68
PA (ng/mL)	6.47 ± 1.55	5.84 ± 1.98	0.95
3-HAA (ng/mL)	6.1 ± 2.3	5.82 ± 2.91	0.63
Trp (nmol/mL)	69.48 ± 15.62	75.93 ± 13.39	0.22

Values are presented as mean ± SD; EPA—eicosapentaenoic acid; DHA—docosahexaenoic acid; 3-HK—3-hydroxykynurenine; KYN—kynurenine; KYNA—kynurenine acid; QA—Quinolinic acid; ; XA—xanthurenic acid; PA—picolinic acid; 3-HAA—3-Hydroxyanthranilic acid; Trp—tryptophan; statistically significant are bold; *—statistically significant difference compared to PRE; *p* < 0.05.

## Discussion

**Figure 3:**
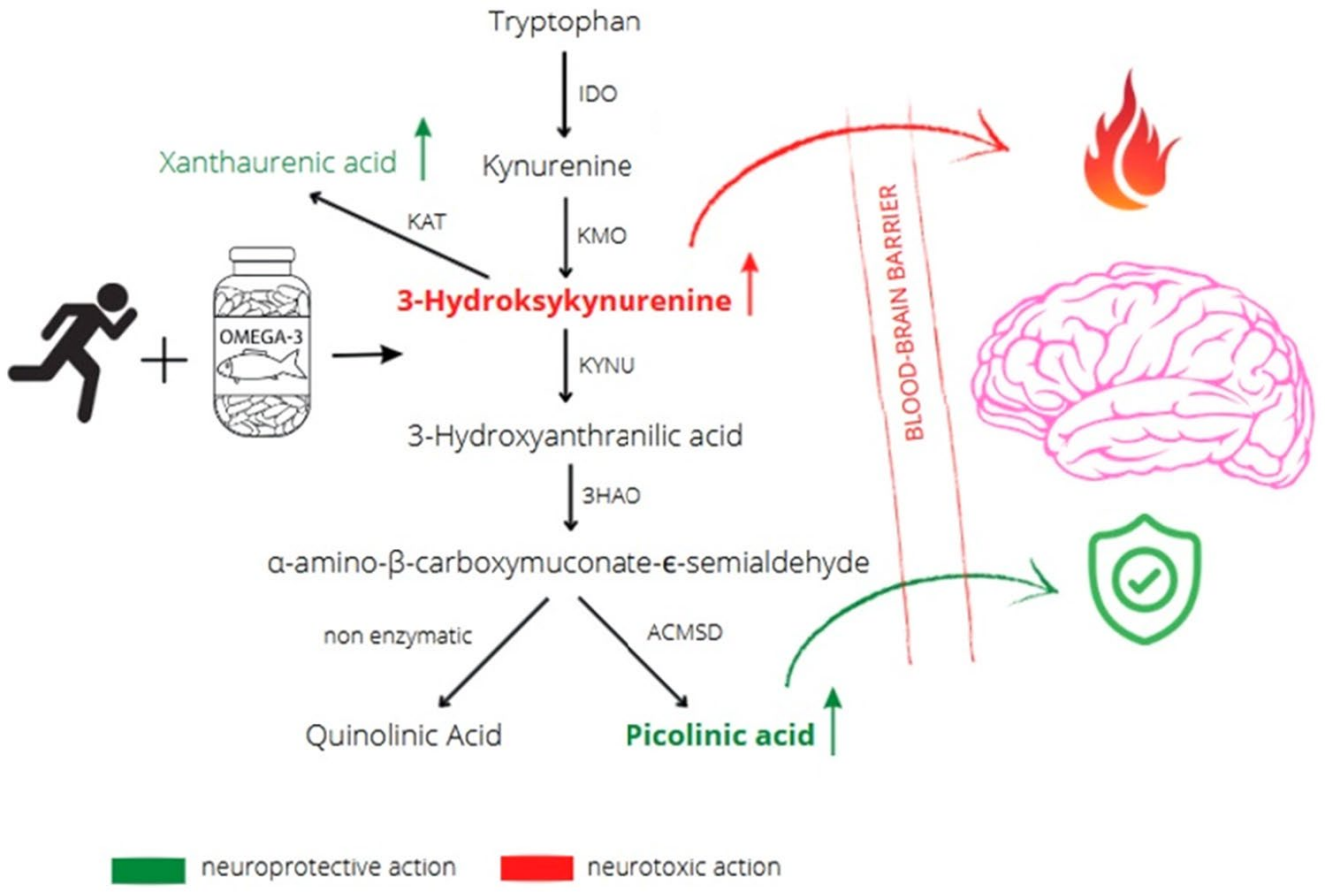
12 weeks of n-3 PUFA supplementation and endurance training increase plasma concentrations of neuromodulating tryptophan metabolites that cross the blood brain barrier (3-hydroxykynurenine, picolinic acid and xanthaurenic acid) in amateur runners. bold letters—statistically significant; regular letters—large or medium group x time interactions. IDO—indoleamine 2,3-dioxygenase; KMO—kynurenine 3-monooxygenase; KAT—kynurenine aminotransferase KYNU—kynureninase; 3HAO—3-hydroxyanthranilate oxidase; ACMSD—aminocarboxymuconate semialdehyde decarboxylase.

The main observation in our study is that 12 weeks of n-3 PUFA supplementation accompanied by endurance training significantly modified metabolism of Trp, specifically increasing plasma concentrations of neurotoxic 3-HK and its neuroprotective metabolite, PA. Studies performed in rodents show that Trp and some of its metabolites like KYN, 3-HK, XA and PA can penetrate the blood–brain barrier which suggests that their blood concentration may contribute to their cerebral pool. Conversely, the ability of QA, KYNA and 3-HAA to cross the blood–brain barrier is low; therefore plasma levels of these metabolites are not expected to significantly influence the brain pools under physiological conditions^13,14^. Significant increase in plasma PA in runners who supplemented with n-3 PUFAs, could be a marker of an adaptive response to exercise due to its established neuroprotective effects in humans^14^ and inhibition of dopamine beta-monooxygenase, which catalyzes the oxidation of dopamine to norepinephrine in animal models^27^. Although not statistically significant, there were also large and medium group x time interactions for KYNA and XA respectively, in athletes who were supplemented with n-3 PUFAs. This is in line with data that formation of XA from 3-HK as well as conversion of KYN to KYNA, catalyzed by KATs, is upregulated by endurance training^28,29^. Therefore, adapted athletes should efficiently convert KYN to KYNA and 3-HK to XA^30^. Moreover, XA has been shown to stimulate a dose-dependent increase in dopamine release in the cortex and striatum of^31^, which together may confirm our hypothesis about beneficial adaptation effects as a result of supplementation n-3 PUFAs. Finally, metabolic pathways of 3-HK and 3-HAA lead to formation α-amino-β-carboxymuconate-ϵ-semialdehyde which can be converted to QA or PA (Fig. 3).

Our results suggest that n-3 PUFA supplementation accompanied by endurance training leads to increased formation of neuroprotective PA but not QA, which exhibits well established neurotoxic properties^32^. Moreover, a decrease in the plasma ratio of neurotoxic to neuroprotective metabolites: 3HK to PA and of KYN + 3HK to XA + PA was observed in runners supplemented with n-3 PUFAs. Similarly, large group x time interactions for KYNA and 3-HAA and a medium group x time interaction for XA were revealed only in O-3 + TRAIN group. Even though not statistically significant, these results may indicate the neuroprotective effect of n-3 PUFAs. No change in Trp to KYN ratio was observed (Fig. 1A), which excludes changes in indoleamine 2,3-dioxygenase (IDO) activity, which catalyzes the conversion of Trp to KYN^12^. Additionally, percentage increase in RBC EPA correlated with the changes in plasma 3-HK, PA, and 3-HAA. Considering the decline in 3-HAA concentrations in a range of neurological disorders^33^ and the paucity of data on its changes as a result of long-term supplementation with n-3 PUFAs in healthy individuals, future studies addressing the link between 3-HAA and neuromodulation seem warranted. To determine association between changes in Trp metabolite concentrations and mental state, mood was assessed at two time points: 1) after the supplementation period 2) after the performance and stress manipulation tests. In both measurements the majority of participants had a positive or a very positive mood. The scores on the three dimensions of the Mood Adjective Check List scale (energetic arousal, tense arousal and hedonic tone) were not statistically different between the O-3 + TRAIN and TRAIN groups. Additionally, no difference was observed between the two time-points in energetic arousal and tense arousal. During the second time-point participants from both groups scored significantly higher on hedonic tone—the trait underlying the characteristic ability to feel pleasure. Such difference did not occur in the control group. Research on endorphin release after physical activity suggests that even a single workout improves mood^34^ which is in agreement with the results we obtained. However, statistical significance between O-3 + TRAIN and TRAIN groups was not found. The increase in plasma concentrations of neuroprotective Trp metabolites we observed could potentially contribute to improved mood of participants after stress induction. Results of randomized control trials have shown that long-term EPA and DHA supplementation ameliorates mood disorders, impulsive behaviour and improves cognitive functions^19^. However, for healthy individuals, results are inconsistent in this field^35–37^. In a study by Giles et al., 4-week supplementation with 1680 mg of EPA + 1120 mg of DHA daily did not affect stress-induced changes in mood in healthy individuals^35^. Similarly, in a 4-week experiment, Antipa et al. showed little effect of n-3 PUFAs on cognitive function and mood states in healthy students using 1740 mg of EPA + 250 mg of DHA daily^36^. In contrast, Fontani et al. observed improvements in mood profile in healthy young adults as an effect of 4-week supplementation of 1600 mg of EPA + 800 mg of DHA daily^37^. As indicated by Giles et al. the inconsistent results on mood and cognitive outcomes after n-3 PUFA supplementation may be partially attributed to differences in the degree to which subjects experienced stress^35^. It has been shown that both n-3 PUFAs and physical training can induce an anti-inflammatory response^4–7^ while pro-inflammatory cytokines upregulate IDO activity and Trp breakdown^38^. Moreover, changes in kynurenines and inflammatory markers due to acute exercise have been shown^28^. Our data do not show significant changes in serum IL-6 during the course of the study, which is in line with other studies taken on young, healthy individuals^39,40^. Thus, changes in Trp metabolites we observed may not be associated with the effects of n-3 PUFA supplementation on inflammation but rather should be treated like its effects on adaptive response to endurance exercises.

## Strengths and limitations

Since there is gradual incorporation of n-3 PUFAs into RBC membranes, long-term supplementation with n-3 PUFAs (at least 12 weeks) must be applied to significantly increase the concentrations of EPA and DHA^41^. In this study a 12-week supplementation was used, which increased the concentrations of EPA and DHA in erythrocytes from inadequate to the recommended values^19^. Simultaneously, this is the first study to evaluate the effect of long-term supplementation with n-3 PUFAs on resting concentrations of plasma Trp and Trp metabolites which suggests that n-3 PUFA supplementation may improve adaptive changes in Trp metabolism when accompanied by endurance training. Nevertheless, there are also some limitations. To date, studies evaluating penetration of Trp metabolites across the blood–brain barrier have been conducted in animal models^13,14^, therefore our results should be treated with caution. Future studies should also consider a larger group of participants to strengthen the reliability of the data. Finally, mood examination should also be applied at the beginning of the intervention which would provide a full picture of its changes.

## Conclusions

In conclusion, our study demonstrates that 12 weeks of n-3 PUFA supplementation accompanied by endurance training significantly modulates resting metabolites of Trp. Increased concentrations of PA, which is a metabolite of Trp with documented positive effects on brain functions in runners supplemented n-3 PUFAs, point to enhancing effects of n-3 PUFAs on the adaptive response to endurance exercises. Our data indicates that plasma PA and possibly other metabolites could be a marker of adaptive response to exercise when accompanied by n-3 PUFA supplementation.

## Data Availability

Data may be available by email to the principal investigator jant@gumed.edu.pl on reasonable request.

## Abbreviations

DHA: Docosahexaenoic acid
EA: Energetic arousal
EPA: Eicosapentaenoic acid
HT: Hedonic tone
3-HK: 3-Hydroksykynurenine
3-HAA: 3-Hydroxyanthranilic acid
IDO: Indoleamine 2,3-dioxygenase
KAT: Kynurenine aminotransferase
KYN: Kynurenine
KYNA: Kynurenic acid
LC–MS/MS: Liquid chromatography with tandem mass spectrometry
MCT: Medium chain triglycerides
UMACL: Mood adjective check list
QA: Quinolinic acid
PUFA: Polyunsaturated acids
RBCs: Red blood cells
TA: Tense arousal
Trp: Tryptophan
PA: Picolinic acid
XA: Xanthurenic acid
TSST: The trier social stress test
